# La connectivite mixte: prévalence et caractéristiques cliniques chez le noir africain, étude de 7 cas au Gabon et revue de la littérature

**DOI:** 10.11604/pamj.2017.27.162.12572

**Published:** 2017-06-30

**Authors:** Landry Missounga, Josaphat Iba Ba, Ingrid Rosalie Nseng Nseng Ondo, Maria Ines Carine Nziengui Madjinou, Doris Malekou, Emeline Gracia Mouendou Mouloungui, Emmanuel Ecke Nzengue, Jean Bruno Boguikouma, Moussavou Kombila

**Affiliations:** 1 Département de Médecine Interne et Spécialités Médicales, Université des Sciences de la Santé, BP 4009 Libreville Gabon; 2 Service de Cardiologie, Centre Hospitalier Universitaire de Libreville, BP 2228 Libreville, Gabon

**Keywords:** Connectivite mixte, prévalence, noir africain, Gabon, Mixed connective tissue disease, prevalence, black African, Gabon

## Abstract

La littérature rapporte que la connectivite mixte semble plus fréquente dans la population noire et chez les asiatiques. Le but de l'étude était de déterminer la prévalence de la connectivite mixte (CM) parmi les connectivites et l'ensemble des pathologies rhumatologiques dans une population hospitalière au Gabon; de décrire ensuite les caractéristiques cliniques de la maladie. Il s'agissait d'une étude rétrospective des dossiers de patients suivis pour connectivite mixte (critères de Kasukawa) et les autres entités de connectivites (critères ACR) en rhumatologie au CHU de Libreville entre janvier 2010 et décembre 2015. Pour chaque cas de CM, les manifestations articulaires et extra-articulaires, le taux d'anticorps anti-U1RNP, l'évolution, étaient les paramètres étudiés. Sept cas ont été colligés en 6 ans parmi 6050 patients et 67 cas de connectivites soit une prévalence de 0,11% et de 10,44% respectivement. Il s'agissait de 7 femmes (100%), d'âge moyen de 39,5 ans. Les signes articulaires comprenaient: polyarthrite, myalgies, doits boudinés et phénomène de Raynaud dans 87,5%, 87,5%, 28,6% et 14% respectivement. Les 7 patients avaient un taux d'anti-U1RNP élevé entre 5 et 35N (N≤7 UI). Un cas de décès par HTAP était constaté. Il s'agit de la série de CM la plus importante rapportée en Afrique noire. La maladie semble rare chez le noir africain, la raison pourrait être génétique. Les aspects démographiques et cliniques paraissent similaires chez les caucasiens, les asiatiques et les noirs hormis une faible fréquence du phénomène de Raynaud chez les noirs.

## Abstract (en)

The literature reports that mixed connective tissue disease seems more frequent in the black population and among Asians. This study aims to determine the prevalence of mixed connective tissue disease (MCTD) among connective tissue disorders and all rheumatologic pathologies in a hospital population in Gabon as well as to describe the clinical features of this disease. We conducted a retrospective study by reviewing the medical records of patients treated for mixed connective tissue disease (Kasukawa criteria) and other entities of connective tissue disorders (ACR criteria) in the Division of Rheumatology at the University Hospital in Libreville between January 2010 and December 2015. For each case of MCTD the parameters studied were articular and extra-articular manifestations, anti-U1RNP antibodies levels, patient's evolution. Over a period of 6 years, data were collected by medical records of 7 patients out of 6050 patients and 67 cases of connective tissue disorders, reflecting a prevalence of 0.11% and 10.44% respectively. the 7 patients were women (100%), with an average age of 39.5 years. Articular manifestations included: polyarthritis, myalgias, chubby fingers and Raynaud's phenomenon in 87.5%, 87.5%, 28.6% and 14% respectively. The 7 patients had high anti-U1RNP antibodies levels, ranging between 5 and 35N (N≤ 7 IU). A case of death due to pulmonary arterial hypertension (PAH) was certified. This is the largest case series of MCTD reported in Black Africa. The disease seems to be rare among the black Africans; the reason could be genetic. The demographic and clinical aspects appear similar to those in Caucasians, Asians and Blacks except for a low frequency of Raynaud?s phenomenon among Blacks.

## Introduction

Depuis 1972, une nouvelle connectivite a été décrite par Sharp et lui a valu son syndrome éponyme [1]. Son polymorphisme clinique, par la coexistence de signes de connectivites différentes chez un même patient, est à l'origine des controverses sur sa reconnaissance comme entité à part entière. La présence constante d'anticorps anti-U1RNP à des taux sériques élevés est l'une des caractéristiques de la connectivite mixte (CM) unanimement reconnue alors que différents critères ont été proposés pour son diagnostic [2–4]. La prévalence précise de la CM reste inconnue. Elle serait plus importante dans la population noire et chez les asiatiques [5]. Les plus grandes séries de la littérature concernent pourtant des populations caucasiennes ou asiatiques [5]. Les études africaines rapportent peu de cas de cette connectivite dans des populations noires. Pour juger de l'importance de la CM chez les sujets noirs d'Afrique, nous avons mené cette étude qui avait pour objectifs: de déterminer la prévalence de la CM parmi les connectivites et l'ensemble des pathologies rhumatologiques dans une population hospitalière au Gabon, de décrire les caractéristiques cliniques de la CM chez des patients du Gabon avant de les comparer aux autres CM noire-africaines, caucasiennes et asiatiques.

## Méthodes

**Cadre, type et période de l'étude:** Nous avons fait une étude rétrospective des dossiers de patients suivis en consultations de rhumatologie au CHU de Libreville entre janvier 2010 et décembre 2015, soit une période de 6 ans.

**Critères d'inclusion:** Les dossiers retenus devaient comporter le diagnostic de connectivite mixte sur la base des critères de Kasukawa [**3**]. Le diagnostic des autres connectivites: lupus érythémateux systémique (LES), sclérodermie systémique (SSc), syndrome de Gougerot-Sjögren (SGS), dermato-polymyosite (DPM), syndrome des anti-phospholipides (SAPL) était basé sur les critères diagnostiques de l'American College of Rheumatologie (ACR) de chaque entité.

**Critères de non inclusion:** Les connectivites indifférenciées n'étaient pas prises en compte.

**Collecte et saisie des données:** Les données étaient collectées dans une fiche standardisée comportant les paramètres étudiés: l'âge et le sexe des patients avec diagnostic de CM, les manifestations articulaires et extra-articulaires, le taux d'anti-U1RNP, le test VIH, les traitements et l'évolution des patients contrôlés tous les 3 mois au cours des 6 années de suivi. La saisie des données étaient réalisée avec le logiciel CSPro dans sa version 6.0. la comparaison des données de notre étude avec celles de la littérature a été faite par revue de la littérature avec les mots « mixed connective tissue disease » dans la base PubMed.

## Résultats

Sept cas de CM ont été colligés parmi 6050 patients rhumatisants en 6 ans, soit une prévalence de 0,11%. La CM était au quatrième rang des connectivites suivies pendant cette période (n=7; 10,4%) derrière le LES, le SGS, la PM, le SAPL et devant la SSc Tableau 1. Dans les sept cas il s'agissait de femmes noires africaines (100%). Les caractéristiques cliniques, immunologiques et évolutives des patients sont rapportées au Tableau 2. L'âge moyen des patients était de 39,5 ans avec des extrêmes à 18 ans et 56 ans. La durée moyenne des symptômes avant le diagnostic de CM était de 16,5 mois avec des délais qui variaient entre 2 mois et 6 ans. Les manifestations articulaires étaient les suivantes: polyarthrite non érosive dans 6 cas (85,7%), arthralgies dans 1 cas (14,3%), myalgies dans 6 cas (85,7%), doigts boudinés dans 2 cas (28,6%), phénomène de Raynaud dans un cas (14,3%). Les signes extra-articulaires comprenaient: une dysphagie par oesophagite, une anémie et la toux dans 2 cas chacune (28,6%), un amaigrissement dans un cas (14,3%). L'anticorps anti-U1RNP était retrouvé à des titres élevés de 5 à 35 fois la normale chez les 7 patientes (100%). Il y avait un taux élevé d'anticorps anti-nucléaires de type moucheté supérieur à 1280 UI et à 640 UI respectivement dans 6 cas (85,7%) et dans un cas (14,3%). Les comorbidités dans cette série étaient une hypertension artérielle chez 2 patientes (28,6%) et un cas d'hépatite virale C. Il n'y avait aucun cas d'infection par le virus de l'immunodéficience humaine (VIH). Les traitements comprenaient la prednisone dans les 7 cas (100%), l'hydroxychloroquine et le méthotrexate dans 5 cas chacun (71,4%), l'azathioprine chez une patiente (14,3%). Quatre patientes (57,1%) prenaient une combinaison thérapeutique de type méthotrexate-hydroxychloroquine (n=3; 42,8%), azathioprine+hydroxychloroquine (n=1; 14,3%). Une des sept patientes a évolué vers un lupus systémique (14,3%) après deux ans de suivi, avec présence d'anticorps anti-Sm et anti-DNA Natif respectivement à 3xN et à 5xN. L'évolution du lupus était favorable avec un indice SLEDAI (systemic lupus erhytematosus disease activity index) passant de 8 à 4 sous prednisone et combinaison azathioprine-hydroxychloroquine après six mois de traitement. Six parmi les 7 patientes (85,7%) avaient une évolution favorable après un suivi moyen 3,8 ans d'une durée allant de 17 mois à 6 ans. Une patiente de la série est décédée par hypertension artérielle pulmonaire, soit une mortalité de 14,3%.

**Tableau 1:** Prévalence des connectivites en Rhumatologie au Gabon entre janvier 2010 et décembre 2015

Connectivites	effectifs N=67	Prévalence %	Fréquence globale /6050 P%
LES	30	44,8	0,49
SGS	10	15	0,16
DM/DPM	8	12	0,13
CM	7	10,4	0,11
SAPL	7	10,4	0,11
SSC	5	7,4	0,08

LES: lupus érythémateux systémique, SGS: syndrome de Gougerot-Sjögren, DM: dermatomyosite, PM: polymyosite, CM: connectivite mixte, SAPL: syndrome des anti-phospholipides, SSc: sclérodermie systémique

**Table 2:** Caractéristiques cliniques, immunologiques et évolutives de 7 cas de connectivité mixte suivis en rhumatologie au Gabon

Patients N=7	Sexe	Age ans	Signes articulaires Extra-articulaires	Ac sériques U1RNP (N˂5 U/ml)	Suivi *évolution*
1	F	54	**Polyarthrite:** myalgies, toux	48,2 x N	M12, *favorable*
2	F	56	**Polyarthrite:** myalgies, Raynaud	32 x N	M36, *favorable*
3	F	18	**Polyarthrite:** myalgies, toux, anémie	9,2 x N	M48, *favorable*
4	F	26	**Arthralgies:** myalgies, anémie	48 x N	M72, *vers un LES*
5	F	29	**Polyarthrite:** myalgies, doigts boudinés	48,2 x N	M24, *favorable*
6	F	42	**Polyarthrite:** oesophagite	5 x N	M48, *favorable*
7	F	52	**Polyarthrite:** Myalgies, doigts boudinés	17 x N	M17, *décès par HTAP*

Ac: anticorps, U1RNP: ribonucleoprotein particle, N: normale, F: féminin, LES: lupus érythémateux systémique, M: mois, HTAP: hypertension artérielle pulmonaire.

## Discussion

Au plan épidémiologique, Les plus grandes séries de CM de la littérature à ce jour sont européennes avec 280, 161,147 et 103 cas en Hongrie, en Italie, en Norvège et en Pologne respectivement [6–9]. Les séries asiatiques viennent de Chine (91 cas) et du Japon (86 cas) [10, 11]. Le registre américain compte 122 cas colligés à la Mayo Clinic de Rochester dans le Minnesota (50 cas), dans le Missouri (51 cas) et à Miami (21 cas) [12, 13]. Dans cette ville considérée comme microcosme de la diversité ethno-raciale des USA, la cohorte de patients avec CM ne comportait que 6 noirs américains alors que dans le groupe contrôle des 39 patients lupiques, il y avait 18 noirs (46%), 1 caucasien (3%) et 20 hispaniques (51,3%) [13]. Ce travail confirme la forte fréquence du LES chez le sujet noir, unanimement reconnue tandis que la CM parait peu fréquente chez les noirs américains. En Afrique noire, le plus grand nombre de cas de CM publié à ce jour est rapporté au Sénégal avec seulement 6 patients en 22 ans dont 2 cas sur 260 connectivites colligées en 10 ans [14–16]. Parmi les 13517 patients rhumatisants colligés en 15 ans au Togo par Houzou P, aucun n'avait une CM [17]. Il en est de même dans les travaux sur les connectivites en milieux hospitaliers au Togo et au Burkina Faso respectivement par Mijiyawa M et Ouedraogo DD [18, 19]. Dans la série Camerounaise de 36 rhumatismes inflammatoires chroniques, Noche CD a recensé 2 cas de CM entre 2004 et 2012 alors que la polyarthrite rhumatoïde et le lupus systémique représentaient 16 et 8 cas respectivement [20]. Daboiko et al ont retrouvé 2 cas de CM parmi 18 cas de connectivites dominés par le LES (10 cas) entre 1998 et 2000 en Côte d'Ivoire [21]. Lutalo SK rapporte aussi 2 cas au Zimbabwe en 1985 avec plutôt un profil d'overlap syndrome [22].

Tous ces travaux suggèrent que la CM est une entité rare chez le sujet noir en général et en particulier chez le noir africain. Dans les années 1980-1990 l'on pouvait incriminer l'insuffisance du plateau technique et la carence en spécialistes rhumatologues ou internistes capables de rechercher les auto-anticorps pour le diagnostic des connectivites dans les pays d'Afrique subsaharienne. De nos jours bien de cas de connectivites telles que le LES, le SGS, la SSc ou les PM/DM sont de plus en plus rapportés en Afrique subsaharienne [18, 19, 23, 24]. La rareté de la CM ne peut donc être attribuée à une difficulté de diagnostic dans la mesure où en pratique la mise en évidence d'un taux sérique élevé d'anticorps anti-U1RNP, indispensable au diagnostic de CM, n'est pas faite isolément. L'anti-U1RNP fait en effet partie des anticorps anti-antigènes solubles des noyaux (ENA ou ECT) dont le dépistage et le titrage permettent le diagnostic des différentes connectivites (anti-Sm pour le LES, anti-SCL70 pour la SSc, anti-SSA/SSB pour le SGS) sans technicité et sans surcoût spécifiques à chaque entité. Force est donc de constater que la CM est bien rare chez le noir africain. L'explication pourrait être génétique. Bien que l'éthiopathogénie de la CM reste peu connue, la susceptibilité de cette entité à l'antigène HLA DR4 ou DR2 (allèles DRB1*0101 ou DRB1*0401) est unanimement reconnue chez le sujet caucasien [9, 25, 26]. A l'image de l'antigène HLA B27 dont la faible fréquence chez les sujets noirs expliquerait la rareté de la spondylarthrite ankylosante en Afrique subsaharienne, l'absence ou une fréquence faible de l'antigène HLA DR4 ou de ses sous-types à risque de la CM dans les populations noires d'Afrique, pourrait être en cause. La susceptibilité de la CM à l'HLA DRB1 a été récemment confirmée dans deux études de cohortes de patients caucasiens en Norvège [24] et en Pologne [25]. Ces travaux ont montré que l'allèle DRB1 était significativement plus fréquent dans la CM que dans les populations contrôles. Ils ont permis d'identifier des sous-types plus à risque de la maladie: DRB1*04 et *09:2 en Pologne, DRB1*04:01 et B*8 en Norvège. Ils ont également identifié des allèles de protection contre la CM: DRB1*07: 01 dans la série Polonaise, DRB1*04 :04 et *13 :01 dans la cohorte Norvégienne. Les sujets noirs porteraient-ils plus fréquemment ces allèles protecteurs que les caucasiens? Le typage HLA de classe I chez tout patient avec connectivite quelle qu'en soit l'entité pourrait apporter des réponses à la participation de facteurs génétiques dans la pathogénie de la CM chez le sujet noir. Dans le registre Norvégien des connectivites, Flam ST et al ont identifié les sous-types *08, *03 :01 et *08 :01 à risque pour le LES, les PM/DM et la SSc respectivement [24]. Le coût de ces examens génétiques reste un obstacle pour juger du lien éventuel entre ces connectivites et le complexe d'histocompatibilité HLA chez les patients des pays d'Afrique noire.

La possibilité d'une association non fortuite de la CM à des facteurs exogènes principalement des infections par le VIH ou par le virus humain lymphotrope T de type I (HTLV-1) est rapportée dans des observations japonaises [27, 28]. Cette hypothèse parait peu probable particulièrement pour le VIH/SIDA dont l'Afrique sub-saharienne est l'une des régions les plus affectées par ce virus. Les pathologies rhumatologiques reconnues associées au VIH dans ces populations sont principalement les spondyloarthrites de types arthrites réactionnelles et le rhumatisme psoriasique [29]. Aucun des 7 patients de notre série et aucun parmi les 6 du Sénégal n'avait une infection par le VIH/SIDA. Il n'y a donc pas de lien établi à ce jour entre la CM et le VIH chez le noir africain. Les données démographiques retrouvées dans notre étude restent conformes aux travaux de la littérature. La prédominance féminine de la CM est constante quelles que soient les études Tableau 2. Tous les cas rapportés en Afrique noire à ce jour concernent des femmes. Au Mexique et en Inde 17 et 16 cas ont été rapportés en 10 ans et 13 ans respectivement, il n'y avait qu'un homme dans chacune de ces cohortes [30, 31]. Les 16 cas colligés par Lawrence en Inde figuraient parmi 441 cas de connectivites dominées par le lupus systémique [31]. Les auteurs ont conclu que la CM était bien plus rare que le LES en Inde; ce qui rejoint les résultats des travaux africains. L'âge moyen au diagnostic de la CM était de 39,5 ans dans notre étude, ce qui rejoint les données de la littérature qui le situe entre 20 et 50 ans [32]. Des formes à début pédiatrique sont cependant de plus en plus rapportées dans la littérature. La série française de Tellier S comporte 19 enfants suivis pendant 3,2 ans en moyenne avec arthralgies et phénomène de Raynaud dans 100% et 84% des cas respectivement [33].

Au plan clinique le phénomène de Raynaud parait rare dans les CM des sujets noirs africains. Il n'y avait qu'un seul cas de Raynaud parmi les 7 cas de notre série et dans celle du Sénégal. Cette manifestation est au premier plan de la maladie et souvent inaugurale chez les patients caucasiens (Tableau 2) ainsi que ceux des autres séries rapportées en Inde, en Arabie Saoudite et en Chine avec 80%, 88% et 94,5% de cas de Raynaud respectivement [10, 31, 34]. Dans le registre Norvégien de CM, c'est la manifestation la plus constante après la positivité de l'anti-U1RNP [8]. Les arthralgies sont un signe constant dans la CM d'où l'intérêt rhumatologique de l'affection. Il s'agit souvent d'arthrites non érosives comme dans notre série, leur association avec un phénomène de Raynaud et des doigts boudinés constitue la triade clinique la plus fréquente de la CM dans la littérature [32]. Dans la récente cohorte Américaine de Ungprasert P, les arthralgies étaient la première manifestation suivie par le Raynaud et les doigts boudinés dans 86%, 80% et 64% des cas respectivement au moment du diagnostic [12]. La fréquence des myalgies semble plus élevée dans les CM Africaines (85,7% dans notre série) comparée aux CM caucasiennes d'Amérique et d'Europe où elle varie entre 24% et 42% [12, 8]. Au plan pronostic un patient de notre série (14,3%) est décédé d'une hypertension artérielle pulmonaire (HTAP). Cette dernière constitue la principale cause de mortalité de la CM dans des proportions variables selon les travaux de la littérature (Tableau 3). Son dépistage est recommandé dès le diagnostic de la CM car elle peut survenir tôt ou tard dans l'évolution de la maladie et souvent sans signe radiographique. Dans la série Norvégienne, la prévalence de l'HTAP est de 3,4% parmi 147 patients suivis en 10 ans. Jugée plus faible qu'attendue, elle est redoutable car mortelle chez 3 des 5 patients dépistés [35]. Dans la série Hongroise vingt-deux décès ont été enregistrés parmi 280 patients suivis entre 1979 et 2011, l'HTAP était la première cause de décès pour 9 des 22 patients [8]. Au plan thérapeutique, Il n'y a pas de schéma de consensus particulièrement recommandé à ce jour dans la CM. La prédominance des arthrites et des myalgies chez nos patients nous a fait recourir à la prednisone chez l'ensemble des patients associée au méthotrexate (MTX) dans 71,4% des cas. Ce dernier a fait preuve de son efficacité sur les arthrites notamment dans l'étude Polonaise de Kowal-Bielecka [36].

**Tableau 3:** Profil démographique, clinique et évolutif de la connectivité mixte chez 7 patients au Gabon comparé à d’autres séries de la littérature

Etudes Auteurs	Effectif (N)	Sexe N (%)		Age moyen (ans)	Signes cliniques (%)			Evolution Mortalité
Pays/période		F	M		Art	Ryn	Myo	%
Hajas A [6] Hongrie/ 1979-2011	280	224 (80)	56 (20)	47,6	65,3	38	35,3	7,8
Gunnarsson R [8] Norvège/1996-2005	147	110 (75)	37 (25)	37,9	79	99	42	7,9
Shi YH [10] Chine/ 1990-2008	91	80 (87,9)	11(12,1)	40,8	78	94,5	46	6,6
Ungprasert P [12] USA/ 1985-2014	50	42 (84)	56 (20)	47,6	65,3	38	35,3	7,8
Maldonado ME [13] USA/ 2005-2007	72 (6)+	58 (80,6)	14(19,4)	39	76	86	38	-
Diallo S [14] Sénégal/ 2008	**3**+	3 (100)	-	46,3	66,7	33,3	-	33,3
Rayes HA [34] Arabie S/ 1982-1999	18	11 (61,1)	7 (38,9)	17,9	67	88	58	17
Notre étudeGabon/ 2010-2015	**7**+	7 (100)	-	39,5	85,7	14,3	85,7	14,3

+ **populations noires**, F: femmes, H: hommes, Art: arthrites, Ryn: phénomène de Raynaud, Myo: myosites,

## Conclusion

La prévalence de la CM est faible chez le noir africain au Gabon et dans les autres pays d'Afrique subsaharienne comparée aux autres connectivites notamment le lupus érythémateux systémique et le syndrome de Goujerot-Sjögren. La raison pourrait être plus d'ordre génétique qu'environnementale. Il n'y a pas de disparités démographiques ou cliniques entre les CM caucasiennes, asiatiques et noires africaines hormis une faible fréquence du phénomène de Raynaud dans ces dernières.

### Etat des connaissances actuelles sur le sujet

- la connectivité mixte ou syndrome de Sharp est de plus en plus reconnue comme entité à part entière grâce aux progrès de la biologie moléculaire;
- Sa prévalence dans les populations blanches et asiatiques se situe entre celle de la DPM, plus rare, et du LES, plus fréquent;
- La littérature rapporte que la maladie serait plus fréquente dans les populations noires.

### Contribution de notre étude à la connaissance

- Notre étude est l'une des premières à évaluer la prévalence de la connectivité mixte dans une population d'Afrique noire;
- Elle montre que la prévalence hospitalière de la maladie est faible voire nulle dans les pays d'Afrique subsaharienne où les connectivites ont été étudiées, pour des raisons génétiques possibles qu'il reste à élucider;
- Elle montre aussi que le profil clinique et évolutif de la maladie est quasi identique dans les populations blanches, asiatiques ou noires.

## Conflits d’intérêts

Les auteurs ne déclarent aucun conflit d'intérêt.
